# Imaging GRPr Expression in Metastatic Castration-Resistant Prostate Cancer with [^68^Ga]Ga-RM2—A Head-to-Head Pilot Comparison with [^68^Ga]Ga-PSMA-11

**DOI:** 10.3390/cancers16010173

**Published:** 2023-12-29

**Authors:** René Fernández, Cristian Soza-Ried, Andrei Iagaru, Andrew Stephens, Andre Müller, Hanno Schieferstein, Camilo Sandoval, Horacio Amaral, Vasko Kramer

**Affiliations:** 1 Nuclear Medicine and PET/CT Center PositronMed, Providencia, Santiago 7501068, Chile; csoza@positronmed.cl (C.S.-R.); hamaral@positronmed.cl (H.A.); vkramer@positronpharma.cl (V.K.); 2 Positronpharma SA, Providencia, Santiago 7501068, Chile; 3 Department of Radiology, Division of Nuclear Medicine and Molecular Imaging, Stanford University, Stanford, CA 94305, USA; aiagaru@stanford.edu; 4 Life Molecular Imaging GmbH, 13353 Berlin, Germany; a.stephens@life-mi.com (A.S.); a.mueller@life-mi.com (A.M.); 5 Formerly Piramal Imaging GmbH, 13353 Berlin, Germany; hanno.schieferstein@gmx.de; 6 Merck Healthcare KGaA, 64293 Darmstadt, Germany; 7 Fundación Arturo López Pérez, Providencia, Santiago 750069, Chile; camilo.sandoval@falp.org

**Keywords:** mCRPC, GRPr, PSMA, [^68^Ga]Ga-RM2, [^68^Ga]Ga-PSMA-11, PET imaging

## Abstract

**Simple Summary:**

Prostate cancer is the most prevalent cancer among men. Patients diagnosed with metastatic, castration-resistant prostate cancer (mCRPC) face a highly aggressive disease and reduced overall survival. For these patients, [^177^Lu]Lu-PSMA-617 has shown promising results. However, this therapy may not benefit patients with low or heterogeneous PSMA expression. The gastrin-releasing peptide receptor (GRPr) is highly expressed in prostate cancer and other cancer cells, and [^177^Lu]Lu-labeled GRPr-ligands have demonstrated good tumor uptake and retention, with minimal uptake in healthy tissues. However, the level of GRPr expression in advanced mCRPC patients remains elusive. In this study, we compared [^68^Ga]Ga-RM2 with [^68^Ga]Ga-PSMA-11 in a Latin American mCRPC cohort to evaluate the clinical utility of [^68^Ga]Ga-RM2 in this group of patients. Although GRPr is overexpressed in the early stages of prostate cancer, our results indicate that in more advanced stages, such as mCRPC, the expression is lower than PSMA.

**Abstract:**

Background: The gastrin-releasing peptide receptor (GRPr) is highly overexpressed in several solid tumors, including treatment-naïve and recurrent prostate cancer. [^68^Ga]Ga-RM2 is a well-established radiotracer for PET imaging of GRPr, and [^177^Lu]Lu-RM2 has been proposed as a therapeutic alternative for patients with heterogeneous and/or low expression of PSMA. In this study, we aimed to evaluate the expression of GRPr and PSMA in a group of patients diagnosed with castration-resistant prostate cancer (mCRPC) by means of PET imaging. Methods: Seventeen mCRPC patients referred for radio-ligand therapy (RLT) were enrolled and underwent [^68^Ga]Ga-PSMA-11 and [^68^Ga]Ga-RM2 PET/CT imaging, 8.8 ± 8.6 days apart, to compare the biodistribution of each tracer. Uptake in healthy organs and tumor lesions was assessed by SUV values, and tumor-to-background ratios were analyzed. Results: [^68^Ga]Ga-PSMA-11 showed significantly higher uptake in tumor lesions in bone, lymph nodes, prostate, and soft tissues and detected 23% more lesions compared to [^68^Ga]Ga-RM2. In 4/17 patients (23.5%), the biodistribution of both tracers was comparable. Conclusions: Our results show that in our cohort of mCRPC patients, PSMA expression was higher compared to GRPr. Nevertheless, RLT with [^177^Lu]Lu-RM2 may be an alternative treatment option for selected patients or patients in earlier disease stages, such as biochemical recurrence.

## 1. Introduction

Prostate cancer (PCa) is the most common cancer in men, with an incidence of approximately 30.7 per 100,000 inhabitants (age standardized) [1]. While the five-year survival rate of localized, low-volume prostate cancer is close to 100%, metastatic, castration-resistant prostate cancer (mCRPC) is a highly aggressive disease with a significantly reduced median overall survival, accounting for 3.8% of all cancer deaths in men [1,2,3]. Although taxane-based chemotherapy and other available treatments can mitigate the effects of the disease, mCRPC can eventually progress, leaving the patients without further treatment options.

Recently, radioligand therapy (RLT) with [^177^Lu]Lu-PSMA-617, targeting the prostate-specific membrane antigen (PSMA), has emerged as a promising treatment for advanced PCa patients. RLT with [^177^Lu]Lu-PSMA-617 has demonstrated a remarkable capacity to improve quality of life and overall survival in most patients with mCRPC [4]. Nonetheless, the evidence indicates that approximately 30% of patients already show progression after the first or second treatment cycle [5,6], which might in part be related to a heterogeneous PSMA expression and low, insufficient absorbed doses in individual lesions.

The gastrin-releasing peptide (GRP) can be found in the nervous system and peripheral tissues, such as the gastrointestinal tract. GRP binds to its receptor (gastrin-releasing peptide receptor (GRPr)), a G-coupled protein from the bombesin family. GRPr is overexpressed in different cancers, such as breast cancer, small-cell lung cancer, and gastrointestinal stromal tumors. However, GRPr is also highly expressed in tumoral vessels of urinary tract cancers, particularly treatment-naïve and recurrent prostate cancer [7,8,9], and during early and advanced stages of PCa [10,11]. Therefore, positron emission tomography (PET) imaging with the synthetic GRPr antagonist [^68^Ga]Ga-RM2 has emerged as a useful tool for biopsy guidance in patients with suspected PCa [12] and for staging and localization of disease in patients with primary PCa and patients with biochemical recurrence (BCR) and negative findings on conventional imaging and evaluation of treatment response [8,13,14,15,16]. Thus both, PSMA and GRPr are relevant diagnostic biomarkers for PET imaging in PCa at different stages of the disease [9,14,17,18,19,20]. Nevertheless, the biological mechanisms underlying PCa progression are complex and PET imaging of PSMA and GRPr might provide different insights into the heterogeneity of the disease. For instance, several studies support the notion that not all prostate cancer lesions present high levels of PSMA expression [20,21,22,23]. Interestingly, some metastases are exclusively detected by GRPr-targeted compounds and others are positive only for PSMA-targeted radiotracers, suggesting a complementary role between PSMA- and GRPR-targeted compounds [19,24,25]. However, determining the expression behavior of GRPr in the advanced stages of PCa remains a challenge. On the other hand, both tracers have renal elimination; however, PSMA presents increased physiological uptake in the liver parenchyma, a feature not observed with RM2. The low hepatobiliary uptake of ^68^Ga-RM2 enables the detection of liver metastasis.

Due to the high GRPr expression in PCa, [^177^Lu]Lu-labeled GRPr-ligands have been proposed as a therapeutic alternative for patients with low PSMA expression. This was exemplified in a proof-of-concept study evaluating the biodistribution and dosimetry of [^177^Lu]Lu-RM2 in mCRPC patients showing good tumor uptake, retention, and rapid clearance from healthy tissues [26]. The low hepatobiliary, salivary, and lacrimal gland uptake might represent an advantage of [^177^Lu]Lu-labeled GRPr ligands currently under development considering the high frequency of xerostomia as an adverse effect in patients under RLT with [^177^Lu]Lu-PSMA-617 [27,28]. Furthermore, given the high expression of GRPr in several cancer types, it is a relevant pan-tumor target for RLT [29,30,31]. However, a drawback for RLT may emerge due to the high uptake in the pancreas, leading to undesired side effects attributed to the high radiation dose. Thus, the pancreas is considered a dose-limiting organ for GRPr-mediated treatment. Nonetheless, preliminary data indicate that the uptake is not persistent and cleared within 24 h [26,27,28], and the pancreas is considered radioresistant [32]. However, the extent of GRPr expression in the advanced stages of PCa remains unclear [33,34], and ongoing investigations are evaluating what criteria are appropriate to select patients for GRPr-targeting RLT [35]. In this study, we compared [^68^Ga]Ga-RM2 and [^68^Ga]Ga-PSMA-11 in a cohort of Latin American patients diagnosed with mCRCP to further understand the potential clinical utility of GRPr-targeting RLT in these patients.

## 2. Materials and Methods

### 2.1. Patient Population

This prospective study was approved by the regional ethics committee board (Servicio de Salud Metropolitano Oriente, ethics committee, permit 26042016) and was conducted following the Declaration of Helsinki, Good Clinical Practices, and Chilean regulations. Seventeen subjects (median 66, IQR 8 years of age) with biopsy-proven mCRPC, rising PSA > 2 ng/mL, Gleason score of 8 to 10, testosterone < 20 ng/mL, a performance status score ECOG of 0–3, without further conventional treatment options, and who have been referred for RLT with either [^177^Lu]Lu-PSMA-617 or [^177^Lu]Lu-RM2, were enrolled in the study and gave written informed consent. Previous treatments included surgery (33%), androgen receptor signaling inhibitors (ARSI) (78%), radiotherapy (RT) (67%), and a combination of systemic therapies (ARSI, RT, and chemotherapy) (33%) (Table 1). Patients had PSA levels of 292 ± 465 ng/mL (range 0.05–1365 ng/mL) measured within 4 ± 3 days (range: 1–7 days) prior to PET imaging. Further blood biomarkers were evaluated as inclusion criteria (alkaline phosphatases > 2.5 upper normal limits in the absence of bone metastases; glutamic-oxaloacetic transaminase (GOT) and glutamic pyruvic transaminase (GPT) < 2.5 upper normal limits and up to 5 times if liver metastases are present; and creatinine clearance ≥ 40 mL/min/1.73 m^2^) for all patients included in the study and prior to the intervention. Exclusion criteria included the inability to sign the informed consent, not complying with the inclusion criteria, severe claustrophobia, or being diagnosed with a malignancy other than adenocarcinoma of the prostate.

### 2.2. Radiotracer Preparation

Production of [^68^Ga]Ga-PSMA-11 and [^68^Ga]Ga-RM2 was performed in accordance with local GMP regulations and using a similar procedure as published previously [14]. Briefly, radiolabeling of [^68^Ga]Ga-PSMA-11 and [^68^Ga]Ga-RM2 was performed using a cassette-based module (Gaia, Elysia-Raytest, Straubenhardt, Germany), PSMA-11, cassettes and reagent kits (Advanced biochemical compounds ABX, Dresden, Germany), RM2 (kindly provided from Life Molecular Imaging, Berlin, Germany), and a 2 GBq ^68^Ge/^68^Ga-generator (iThemba Labs, Somerset West 7129, Cape Town, South Africa).

The eluted gallium-68, was trapped on a strong cation exchange cartridge, rinsed with ultrapure water, and eluted with 450 μL eluent (5 M NaCl in 5.5 M HCl) into a mixture of 40 μL precursor (1 mg/mL in ultrapure water) in 3.85 mL buffer (0.08 M ammonium acetate, pH 4.5) and 200 μL ethanol. After radiolabeling at 95 °C for 8 min., the reaction mixture was diluted with 5 mL water. The crude product was extracted using a C18 cartridge and rinsed with water. The purified product was eluted with 1.5 mL 60 vol% ethanol followed by 8.5 mL saline and passed through a 0.22 µm sterile filter (Millex-GV, Merck Millipore, Darmstadt, Germany). Quality control was performed, including controls for visual inspection, pH, radiochemical purity by HPLC, radionuclidic identity, residual solvents, endotoxins, filter integrity (prior release), and sterility (post-release).

### 2.3. PET/CT Imaging and Analysis

All subjects had two PET/CT scans performed on separate days using a Biograph mCT Flow scanner (Siemens Healthineers, Erlangen, Germany) within 8.8 ± 8.6 days (range 1–33 days), without any medical intervention between the scans. The order of [^68^Ga]Ga-PSMA-11 and [^68^Ga]Ga-RM2 PET scans was random and according to the availability of the radiotracer. A contrast-enhanced CT scan and low-dose CT scan were performed for anatomical localization and attenuation correction prior to [^68^Ga]Ga-PSMA-11 and [^68^Ga]Ga-RM2 PET scans, respectively. PET/CT images were acquired head-to-mid-thigh at 60 ± 5 min post-injection of 191 ± 25 MBq (range 122–229 MBq) [^68^Ga]Ga-PSMA-11 and 166 ± 39 MBq (range: 63–243 MBq) [^68^Ga]Ga-RM2, respectively, starting at the pelvis.

Volumes of interest (VOIs) were drawn around tumor lesions, visually distinguished as regions of increased radiotracer uptake relative to adjacent background uptake and outside areas of expected physiological radiotracer uptake. To perform semi-quantitative analysis, mean and maximum standard uptake values (SUVmean and max, respectively) were calculated using Siemens SyngoVia software (SV60). Tumor-to-background ratios (TBRs) were calculated by dividing the SUVmax of different tumor lesions by the SUVmean of the blood pool in the left ventricle of the heart.

### 2.4. Statistical Analysis

The normal distribution of continuous variables was determined with Q-Q plots and histograms. In the case of non-parametric quantitative data, the Wilcoxon signed-rank test was used to compare SUVmax values and TBRs between scans. The test was two-sided, and a *p*-value < 0.05 was considered statistically significant. All statistical analyses were performed using R software version 4.2.0 (22 April 2022) [36]. The sample size for the study considered a minimum of 16 patients (17 were finally included). The sample size calculation was based on the difference between means and standard deviations of SUVmean ratios to the normal background (blood pool) for both tracers (9.2 ± 7.2 for [^68^Ga]Ga -PSMA-11 and 5.2 ± 3.5 for [^68^Ga]Ga-RM2), reported by Minamimoto et al. (2016). The calculation considered a confidence of 95% and a power of 80%, with a correlation of 60%. The analysis was performed using G*power [37].

## 3. Results

### 3.1. Patient Characteristics

Seventeen participants (65.3 ± 7.4 years of age; range: 53–76 years) were enrolled (Table 1) and both PET/CT scans were performed 8.8 ± 8.6 days (range: 1–33 days) apart.

### 3.2. Uptake Comparison between [^68^Ga]Ga-PSMA-11 and [^68^Ga]Ga-RM2

**Figure 1:**
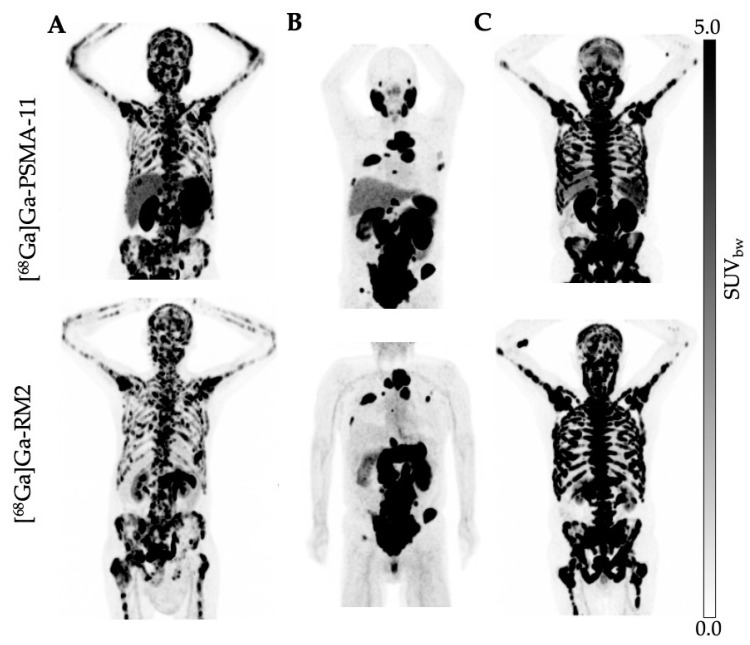
(**A**–**C**) Maximum-intensity projections (MIP) of [^68^Ga]Ga-PSMA-11 (**upper row**) and [^68^Ga]Ga-RM2 (**lower row**) PET images of patients with similar biodistribution and tumor uptake (*n* = 14).

**Figure 2:**
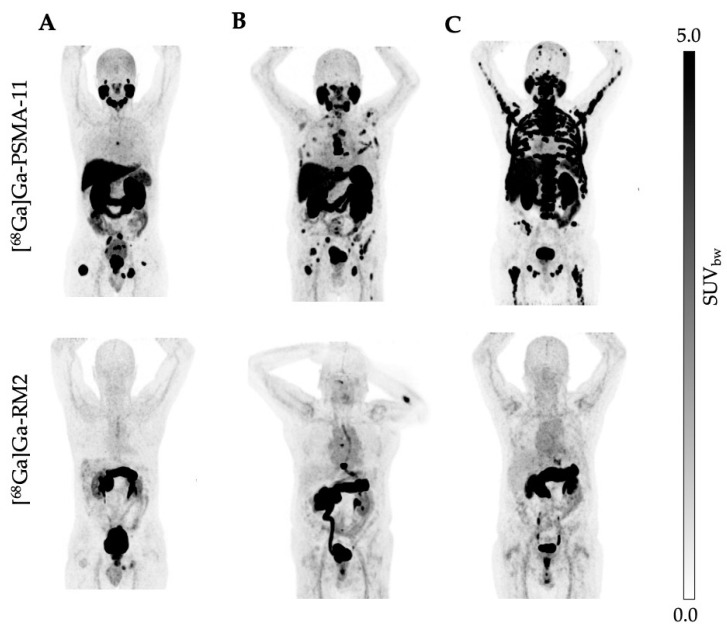
(**A**–**C**) Maximum-intensity projections (MIP) of [^68^Ga]Ga-PSMA-11 (**upper row**) and [^68^Ga]Ga-RM2 (**lower row**) PET images of patients with high PSMA but low GRPr expression (*n* = 13).

The administration of [^68^Ga]Ga-PSMA-11, [^68^Ga]Ga-RM2, and the imaging procedure were well tolerated and no adverse events, discomfort, or change in vital signs was observed. The excretion profile of both tracers was similar with a predominant renal clearance via the kidneys observed for [^68^Ga]Ga-PSMA-11 and [^68^Ga]Ga-RM2 (Figure 1 and Figure 2). However, we observed differences in the physiological biodistribution between the two tracers in the submandibular, parotid, and lacrimal glands, liver, and small intestine, where unlike [^68^Ga]Ga-PSMA-11, [^68^Ga]Ga-RM2 showed no uptake. In contrast, [^68^Ga]Ga-RM2 showed high uptake in the pancreas, whereas no uptake of [^68^Ga]Ga-PSMA-11 was observed.

**Figure 3:**
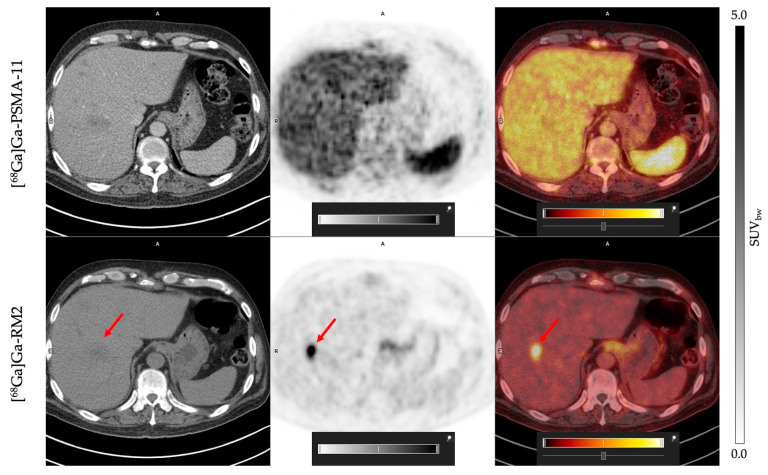
Patient 13: PET/CT [^68^Ga]Ga-PSMA-11 (**upper row**) and [^68^Ga]Ga-RM2 (**lower row**). Red arrows indicates a liver metastasis visible with [^68^Ga]Ga-RM2 and not detected with [^68^Ga]Ga-PSMA-11.

[^68^Ga]Ga-RM2 presents an absence of physiological uptake in the liver, contrary to what was observed with [^68^Ga]Ga-PSMA-11, which favors the detection of possible hepatic metastasis lesions. This was validated in one patient, who exhibited a hepatic lesion with [^68^Ga]Ga-RM2 that was not visible on [^68^Ga]Ga-PSMA-11 scan (Figure 3).

**Figure 4:**
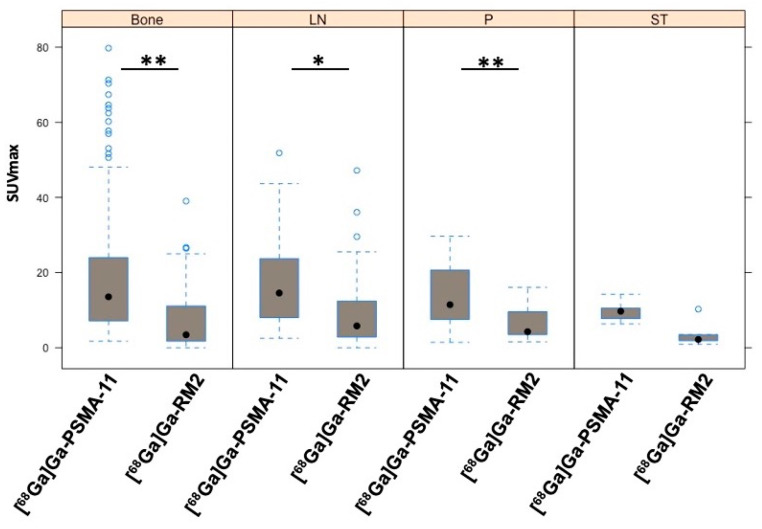
Average SUVmax values of [^68^Ga]Ga-PSMA-11 and [^68^Ga]Ga-RM2 in bone, lymph node (LN), prostate (P), and soft tissue (ST) lesions across all patients. * *p* < 0.05, ** *p* < 0.01. Light blue dots represent outlier values. Black dots represent median values.

Specific uptake in tumor lesions in the prostate, lymph nodes, bone, and soft tissue was evident with both radioligands; however, the SUVmax values of [^68^Ga]Ga-PSMA-11 were statistically higher compared to [^68^Ga]Ga-RM2 in most lesions. Indeed, only 23.5% of the patients showed a high GRPr expression (Figure 1, Figure 2 and Figure 4).

As for the SUVmax values, the same trend was observed when evaluating TBRs for bone, lymph node, prostate, and soft tissue lesions, which were significantly higher for [^68^Ga]Ga-PSMA-11 compared to [^68^Ga]Ga-RM2 (Table 2).

Next, we analyzed whether both tracers were able to detect the same number of lesions considering the total number of lesions found in each patient. In line with our previous results, [^68^Ga]Ga-PSMA-11 detected 23.2% more tumor lesions compared to [^68^Ga]Ga-RM2.

## 4. Discussion

Prostate cancer, and in particular mCRPC, shows high levels of PSMA expression which also correlates with disease stage and severity [38,39]. However, due to the unstable genomic nature of cancerous cells, a tumor may present a great variability of PSMA expression levels resulting in different grades of malignancy and outcomes [40]. For instance, results from the Vision Trial indicate that 50–60% of patients with mCRPC respond with a PSA decline of >50% and an improvement in their overall survival of 15.3 months compared to 11.3 months in standard care. Likewise, imaging-based progression-free survival is also increased in those patients compared to standard care (median, 8.7 vs. 3.4 months) [4,5]. However, approximately 30–40% of mCRPC patients do not respond to [^177^Lu]Lu-PSMA therapy [5,6], which might be due to a heterogeneous PSMA expression or a decrease in PSMA triggered by an aggressive trans-differentiation process, resulting in cancerous cells resistant to therapies. Typically, these patients display visceral metastasis, and adenocarcinoma features are reduced or lost [41,42]. This variance or decrease in PSMA expressions affects the patient selection process and subsequently results in low absorbed doses in individual tumor lesions, ultimately reducing the therapeutic efficacy of [^177^Lu]Lu-PSMA therapy [40,43]. Variability in PSMA expression might depend on many different factors. For example, inflammation NF-κB has been involved in resistance to ADT, contributing to mCRPC progression [44]. Signaling pathways such as PI3K/AKT influence the tumor niche inducing different downstream events, including the expression of the H19 gene [45,46] and hypoxia [47]. The interaction between hypoxia and other pathways is, however, complex. The evidence suggests that hypoxia drives transdifferentiation toward an NE-like phenotype promoting tumor resistance [48].

Similar to PSMA, GRPr is a membrane-bound tumor biomarker, which is found to be overexpressed in 84% of prostate cancer cells [49]. While expression of both PSMA and GRPr is increased in prostate cancer cells, the underlying biological mechanisms responsible for this abnormal behavior are distinct. Previous work has shown that in androgen–dependent prostate cancer xenografts, GRPr is highly expressed, but this expression is drastically reduced after castration. These findings suggested that the expression of GRPr may be regulated by the action of androgen [50] and therefore associated with earlier phases of the disease. In contrast, PSMA expression is higher in later and poorly differentiated stages of the disease [51], suggesting inverse expression profiles of GRPr and PSMA. In a pilot study including six biochemically recurrent prostate cancer patients, Minamimoto et al. (2016) compared [^68^Ga]Ga-PSMA-11 with [^68^Ga]Ga-RM2, revealing distinctive biodistribution patterns for both tracers. However, in tumoral tissue, the study concluded that there were no significant differences in uptake between the two tracers [14].

More recently, Minamimoto et al. (2018) demonstrated a detection rate of approximately 72% using [^68^Ga]Ga-RM2 in a prospective study including 32 patients with biochemical recurrence of prostate cancer and negative findings on conventional imaging [15]. In addition, other reports have shown the expression of GRPr in metastatic lymph nodes, bones, and advanced tumor stages [11,52], suggesting the clinical potential of GRPr as a target for PET imaging and RLT and as an alternative to PSMA.

Consequently, the objective of this study was to compare the uptake and performance of [^68^Ga]Ga-RM2 and [^68^Ga]Ga-PSMA-11, with the aim of evaluating their potential as therapeutic targets for RLT in patients with advanced mCRPC.

We and others have shown that PSMA is highly expressed in prostate tumoral lesions and also in kidneys, spleen, lacrimal, parotid, and submandibular glands, small intestine, and bladder [14,40,53]. This is consistent with what we observed in the present study. The physiological expression of GRPr shows a different pattern compared to PSMA and is high in the pancreas, bladder [14], lymph node metastases, and bone lesions of prostate cancer [11]. Interestingly, the low uptake of [^68^Ga]Ga-RM2 in hepatic tissue allowed the detection of a malignant lesion in the liver, while this lesion was not observed in the [^68^Ga]Ga-PSMA-11 PET/CT scan (Figure 4). This observation is in line with results reported by Verhoeven et al. (2023) [54].

In our study, [^68^Ga]Ga-PSMA-11 outperformed [^68^Ga]Ga-RM2 in terms of lesion detection rate, uptake, and imaging contrast in tumor lesions in bone, lymph nodes, and prostate in patients with advanced mCRPC. The SUVmax values for [^68^Ga]Ga-PSMA-11 were significantly higher than for [^68^Ga]Ga-RM2 in most lesions (Table 2). We obtained the same results using tumor-to-background ratios, allowing for the standardization of the image analysis, providing reproducible, consistent and accurate data across different PET scanners and patients [55,56]. Although both tracers detected tumoral lesions in each patient, [^68^Ga]Ga-PSMA-11 detected 23.2% more lesions than [^68^Ga]Ga-RM2. Both tracers show a high affinity for their targets and it is unlikely that the lower detection rate and uptake values of are related to differences in affinity (Ki = 9.3 nM for [^68^Ga]Ga-RM2 and Ki = 7.5 ± 2.2 nM for [^68^Ga]Ga-RM2, respectively, [57,58]). Furthermore, several reports have shown that GRP derivatives present a high affinity to GRPr, demonstrating its potential in clinical applications [7,57,59].

Interestingly, in some patients with advanced disease, both tracers showed a similar biodistribution in tumor lesions (Figure 1). For those patients, alternating cycles between PSMA- and GRPr-targeted RLT may lead to the same treatment response but with less toxicity from each drug. The expression of PSMA in some healthy tissues, such as salivary and lacrimal glands, the kidney, and bone marrow, produces temporary side effects. Our studies, alongside others, have demonstrated that hematological side effects such as pancytopenia are transient and mainly limited to grade 2. Commonly, patients treated with PSMA-targeted RLT experience xerostomia, fatigue, and nausea [60]. In contrast, GRPr-targeted RLT does not affect salivary or lacrimal glands, and the first-in-human dosimetry study has reported that the treatment was well tolerated and showed no side effects. The most intensive uptake, however, is in the pancreas, which is considered a critical organ. Nonetheless, akin to other RLTs with Lutetium-177, the bone marrow is acknowledged as a critical organ, and no significant differences with PSMA-targeted therapies have been noted [26]. Nevertheless, for mCRPC the clinical benefit of using [^177^Lu]Lu-RM2 is limited to patients who have high expression of GRPr and experienced xerostomia as a dose-limiting event after PSMA-targeted RLT.

Previous works have suggested that GRPr expression is higher in initial disease stages and that [^68^Ga]Ga-RM2 may be particularly valuable for detecting well-differentiated, slow-growing prostate cancer lesions [14,34,49,51]. This is further supported by several head-to-head comparison studies with [^68^Ga]Ga-RM2 and [^68^Ga]Ga-PSMA-11 in preoperative intermediate and high-risk PCa and biochemical recurrent PCa where both tracers performed equally [8,24]. Furthermore, a recent clinical trial showed that [^68^Ga]Ga-RM2 is a promising PET tracer to improve the characterization of patients and guide biopsy, particularly in intermediate-risk patients with intraprostatic prostate cancer [61].

While these results support the notion that GRPr expression is reduced in most cases of advanced mCRPC, individual patients with low PSMA but high GRPr expression may still find benefit in GRPr-targeted RLT. In fact, we have recently published a case report series with clinical results of 4 patients included in our study (4 out of 17, 23.5%) who showed high [^68^Ga]Ga-RM2 uptake and were subsequently treated with a single dose of 5.6 GBq [^177^Lu]Lu-RM2. The 3D SPECT/CT and planar images revealed high tumor uptake and stable binding for up to seven days. In this report, we showed that [^177^Lu]Lu-RM2 uptake in pancreatic tissue was high but showed a rapid clearance after 24–48 h. Furthermore, there were no significant differences between baseline levels of red blood cells, leukocytes, platelets, creatinine, or amylase levels pre-therapy and after 1, 4, and 8 weeks of therapy. Two patients showed a partial response during the initial weeks, and no adverse effects were observed, demonstrating the feasibility of [^177^Lu]Lu-RM2 RLT [62]. These results align with the study conducted by Kurth et al. (2019). In their study, 35 patients with mCRPC without further treatment alternatives were imaged using [^68^Ga]Ga-RM2. Subsequently, four patients were selected to receive [^177^Lu]Lu-RM2 treatment. The therapy was well tolerated by all patients, and no side effects were evident. Most of the [^177^Lu]Lu-RM2 uptake was observed in the pancreas where GRPr expression is high. However, due to the rapid clearance of the radiotracer from this organ, the mean absorbed dose for the pancreas was low [26]. Thus, [^177^Lu]Lu-RM2 therapy was considered to be safe and tolerable for mCRPC patients without any other treatment options.

A very recent clinical study (LuTectomy Trial) investigated the use of [^177^Lu]Lu-PSMA-617 as neo-adjuvant therapy prior to radical prostatectomy in patients with localized, high-risk prostate cancer [63]. The study evaluated the dosimetry and safety of [^177^Lu]Lu-PSMA-617 in this indication and the evaluation of long-term oncological benefits is ongoing and might result in a prolonged time until recurrence. Likewise, considering the high expression of GRPr in early stages of prostate cancer, this could also be a potential, clinical indication for [^177^Lu]Lu-RM2.

## 5. Conclusions

In conclusion, our results confirm previous reports [11,14,34,49] that although PSMA and GRPr are both expressed in mCRPC, GRPr expression is reduced in advanced mCRPC patients and [^68^Ga]Ga-PSMA-11 shows significantly higher uptake compared to [^68^Ga]Ga-RM2. Nevertheless, the low physiological uptake of [^68^Ga]Ga-RM2 in the liver allowed the detection of a hepatic lesion in one patient that was not observable with [^68^Ga]Ga-PSMA-11. GRPr-targeted RLT remains a therapeutic alternative for those patients who have limited treatment options and exhibit high GRPr expression. This might include different oncologic indications such as PCa, mCRPC, and lung and breast cancer.

## Figures and Tables

**Table 1:** Patient characteristics, previous treatments, and PET findings in the prostate (P), lymph nodes (LN), bone (B), and soft tissue (ST).

Patient No.	Age (y)	Gleason Score	PSA (ng/mL)	Previous	[^68^Ga]Ga-PSMA-11 *	[^68^Ga]Ga-RM2	Delay
				Treatments			(Days)
1	63	NA	NA	QT + RT + ARSI	B + P	B + P	1
2	65	NA	1206	RT + ARSI	B + LN + P + ST	B + LN + P	2
3	71	NA	7.94	S + QT + RT + ARSI	LN	LN	14
4	53	7	NA	ARSI	LN + B + P	LN + B + P	1
5	76	NA	88.4	S + RT + ARSI	B	B	18
6	54	7(4 + 3)	470	ARSI	B + LN + P + ST	B + LN + P + ST	1
7	75	NA	NA	QT + RT + ARSI	B + P	B + P	7
8	73	NA	660	QT + RT + ARSI	B + LN + P	B + LN + P	2
9	53	8	1	RT **	P + LN	P	6
10	70	6	7.11	NA	P + LN	P + LN	14
11	68	NA	40.1	S + QT + RT + ARSI	B + LN + P	P	14
12	64	NA	1365	RT + ARSI	B + LN + P + ST	B + LN	3
13	55	8	0.05	S + RT + ARSI	B + LN + ST	B + LN + ST	18
14	71	NA	NA	NA	LN + P	P	6
15	66	NA	79.37	S + ARSI	B	B	4
16	64	4 + 3	3.6	S + RT + ARSI	B	B	33
17	71	NA	18	QT + RT + ARSI	B	NL	6

* All patients had metastatic disease at the moment of the study. ** The patient rejected ARSI therapy due to personal reasons. NA: not available.

**Table 2:** SUVmax values and TBRs for [^68^Ga]Ga-PSMA-11 and [^68^Ga]Ga-RM2 in bone, lymph node, prostate, and soft tissue lesions.

Region	Parameter	[^68^Ga]Ga-PSMA-11	[^68^Ga]Ga-RM2	*p*-Value
Bone	SUVmax	17.0 ± 5.2	11.0 ± 5.9	0.0029
	TBR	15.9 ± 10.9	4.3 ± 5.4	0.0023
LN	SUVmax	15.7 ± 10.7	3.5 ±6.0	0.028
	TBR	16.0 ± 12.4	5.7 ± 5.2	0.038
Prostate	SUVmax	16.8 ± 12.2	4.8 ± 4.2	0.002
	TBR	16.5 ± 13.0	5.9 ± 4.6	0.002
Soft tissue	SUVmax	9.8 ± 3.2	1.7 ± 2.1	0.06
	TBR	13.7 ± 11.4	1.5 ± 1.7	0.11

TBR: tumor-to-background ratio. LN: lymph node.

## Data Availability

The data presented in this study are available on request from the corresponding author. The data are not publicly available due to restrictions regarding the privacy of the patients and ethical reasons.
